# Supplementary material for: Blockade of VEGF-C signaling inhibits lymphatic malformations driven by oncogenic PIK3CA mutation
Source: Nat Commun. 2020 Jun 8;11:2869. doi: 10.1038/s41467-020-16496-y (PMC7280302; doi:10.1038/s41467-020-16496-y)
Supplement: Supplementary file 1 — Supplementary Information [file 41467_2020_16496_MOESM1_ESM.pdf]

Supplementary Information

Blockade of VEGF-C signaling inhibits lymphatic malformations driven by oncogenic *PIK3CA* mutation

Martinez-Corral et al.

Supplementary Tables

Supplementary Table 1. Mouse models of *PIK3CA*^{H1047}-driven LM used in the study.

	**Model**	**Cre strain**	**Induction (stage; dose; route)**	**Effect**	**Vessel phenotype**
Embryonic	Macrocystic LM	*Vegfr3-CreER*^{T2}	Early embryonic (E11);<br>2 mg 4OHT;<br>i.p. (pregnant female)	Systemic	Isolated cysts in the cervical (and lumbar) skin; areas of vessel hypersprouting in other regions of the skin
	Microcystic LM	*Vegfr3-CreER*^{T2}	Late embryonic (E14);<br>2 mg 4OHT;<br>i.p. (pregnant female)	Systemic	Lymphatic vessel hyperbranching in all regions of the skin
Postnatal	Acute microcystic LM	*Vegfr3-CreER*^{T2}	Postnatal (P10) with analysis within 7-10 days after induction;<br>100 µg 4OHT;<br>topical (ear)	Systemic	Induction of new lymphatic vessel sprouts
	Localized microcystic LM	*Prox1-CreER*^{T2}	Early postnatal (P2-P12);<br>0.5-2 µg 4OHT;<br>topical (ear)	Local - treated ear	Isolated microcystic lesions; allows studying advanced (isolated) lesions
	Progressive microcystic LM	*Vegfr3-CreER*^{T2}	Post-wean (3 w);<br>100 µg 4OHT;<br>topical (ear)	Systemic	Progressive lymphatic vessel hypersprouting and hyperplasia; robust and reproducible response

Supplementary Table 2. List of primary antibodies.

Application	Antibody	Company and catalog number	Dilution
IF (whole-mount)	hamster anti-mouse PDPN	Developmental Studies Hybridoma Bank, 8.1.1-a	1/200
	rat anti-mouse PECAM1	BectonDickinson, cat 553370	1/200
	rabbit anti-mouse LYVE1	Reliatech, cat 103-PA50AG	1/500
	rat anti-mouse EMCN	Santa Cruz Biotechnology, cat sc-65495	1/200
	goat anti-mouse NRP2	R&D Systems, cat AF567	1/200
	goat anti-mouse VEGFR2	R&D Systems, cat AF644	1/200
	goat anti-mouse VEGFR3	R&D Systems, cat AF743	1/200
	rabbit anti-PROX1	Generated against human Prox1 C-terminus (567-737aa)[14]	1/200
	rabbit anti-GFP	Thermo Fisher Scientific, cat A11122	1/200
	chicken anti-GFP	Abcam, cat ab13970	1/500
	rabbit anti-pS6 (Ser240/244)	Cell Signaling Technology, cat 2215	1/200
	goat anti-mouse VE-cadherin	R&D Systems, cat AF1002	1/200
IF (cells)	goat anti-VE-cadherin	Santa Cruz Biotechnology, cat sc-6458	1/100
	AF568 Phalloidin	Thermo Fisher Scientific, cat A12380	1/100
	rabbit anti-PROX1	Generated against human Prox1 C-terminus (567-737aa)[14]	1/200
	goat anti-mouse VEGFR3	R&D Systems, cat AF743	1/200
Paraffin sections (mouse)	goat anti-mouse VEGFR3	R&D Systems, cat AF743	1/300
	rabbit anti-mouse LYVE1	Reliatech, cat 103-PA50AG	1/200
	rat anti-mouse EMCN	Santa Cruz Biotechnology, cat sc-65495	1/200
	rat anti-mouse CD45 antibody [I3/2.3]	Abcam, cat ab25386	1/200
	anti-mouse TER-119 AF647	BioLegend, cat 116218	1/200
Paraffin sections (human)	mouse anti-human VEGFR3 (9D9F9)	Millipore, cat MAB3757	1/200
	anti-Actin, α-Smooth muscle Cy3	Sigma, cat A2547	1/300
	rabbit anti-human CD45	Sigma, cat HPA000440	1/100
	rabbit anti-human LYVE1	Reliatech, cat 102-PA50AG	1/100
	rabbit anti-human PROX1	Proteintech Group, cat 51043-1-AP	1/100
	mouse anti-human PDPN (D2-40)	Dako-Agilent, cat M361901-2	1/100
FACS (mouse)	rat anti-mouse CD16/CD32	eBioscience, cat 14-0161-85	1/100
	PDPN (8.1.1, PE)	eBioscience, cat 12-5381-81	1/300
	CD31/PECAM1 (390, PE-Cyanine7)	eBioscience, cat 25-0311-82	1/300
	CD45 (30-F11, PerCP-Cyanine5.5)	eBioscience, cat 45-0451-82	1/50
	CD11b (M1/70, PerCP-Cyanine5.5)	eBioscience, cat 45-0112-82	1/50
	PDPN (8.1.1, PE-Cyanine7)	eBioscience, cat 25-5381-82	1/300
	CD31/PECAM1 (390, PerCP-eFluor 710)	eBioscience, cat 46-0311-80	1/100
	CD45 (30-F11, eFluor 450)	eBioscience, cat 48-0451-82	1/50
	CD11b (M1/70, eFluor 450)	eBioscience, cat 48-0112-82	1/50
	Ki67 (SolA15, eFluor 660)	eBioscience, cat 50-5698-80	1/100
	CD45 (30-F11, PerCP)	BD Pharmingen, cat 557235	1/50
	CD11b (M1/70, APC)	eBioscience, cat 17-0112-82	1/100
	F4/80 (BM8, FITC)	BioLegend, cat 123108	1/50

Supplementary Figures

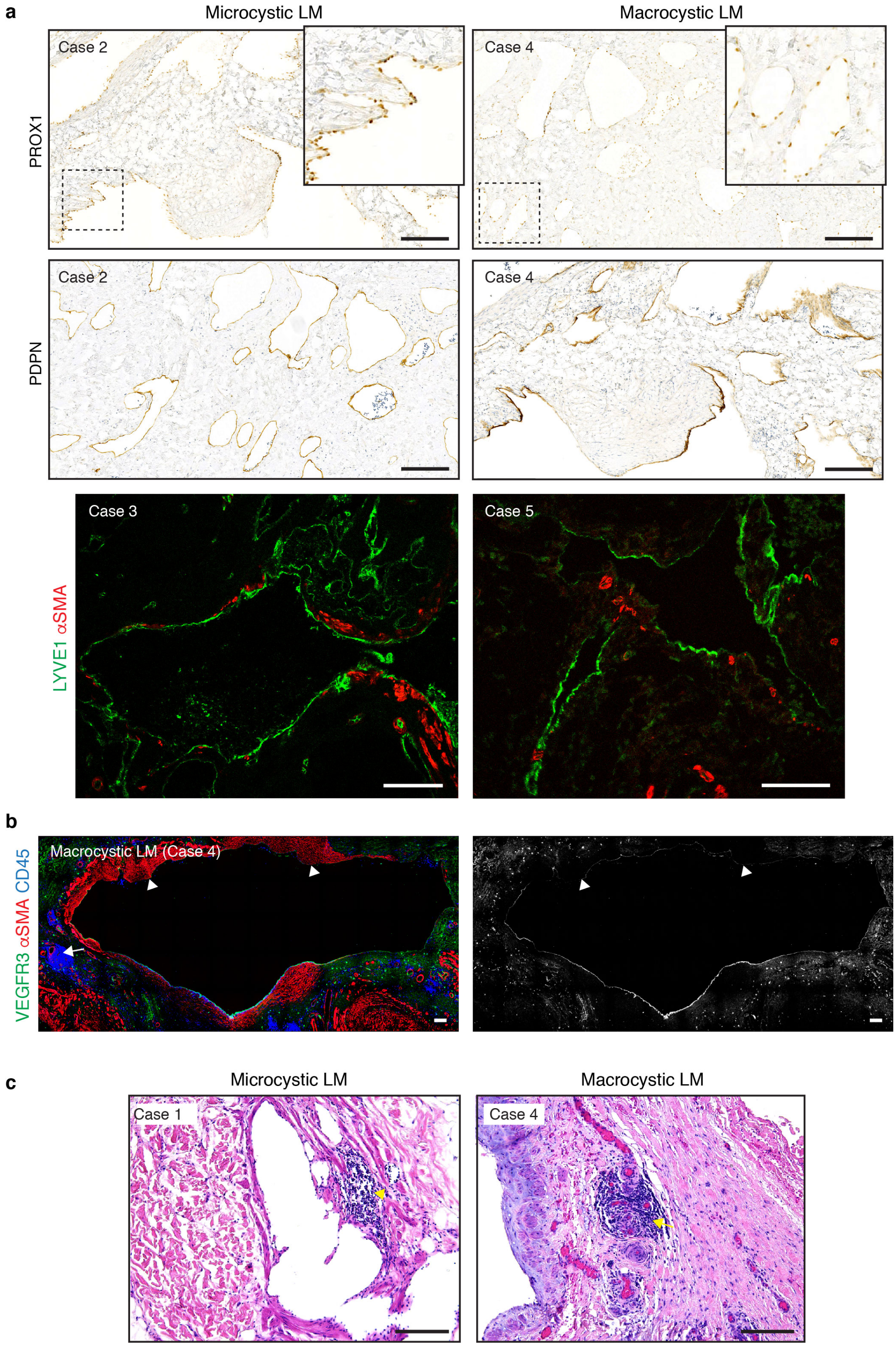

Supplementary Figure 1. Characterization of *PIK3CA^{H1047R}*-driven macrocystic and microcystic LM. a Immunostaining of microcytic (on the left) and macrocystic (on the right) LMs, showing expression of LEC markers PROX1, PDPN and LYVE1. Note heterogeneous expression of LYVE1. **b** Immunofluorescence of a macrocystic LM showing low VEGFR3 expression in cyst endothelium associated with αSMA$^+$ smooth muscle cells (arrowheads) and presence of CD45$^+$ immune cell aggregates (arrow)**. c** Hematoxylin and eosin stained section

of a microcytic (on the left) and a macrocystic (on the right) LMs. Note lymphoid cell infiltration (yellow arrows) and aggregates (yellow arrowhead). Images in (**a**, **b**) are representative of *n*=3 microcystic LM (except for PROX1 two lesions were analyzed) and *n*=2 macrocystic LM lesions. Scale bars: 100 µm (**a**), 200 µm (**b**, **c**).

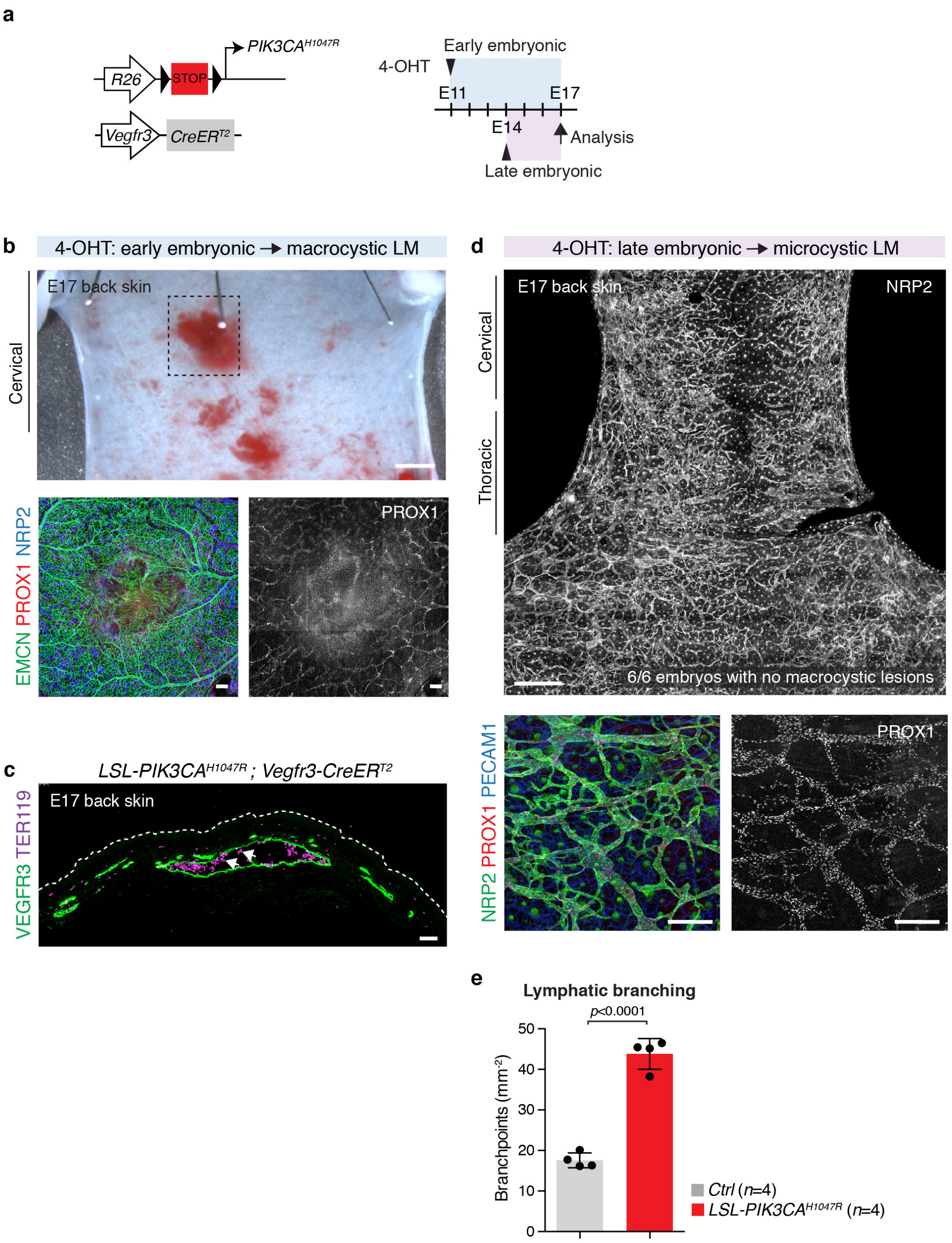

Supplementary Figure 2. Embryonic timing of activation of *PIK3CA* determines LM phenotype. a Genetic constructs and experimental plan for tamoxifen-inducible activation of *PIK3CA*^{H1047R} expression in lymphatic endothelia at early (E11, blue) or late (E14, purple) embryonic development. **b** 4-OHT (2 mg) administration at E11 leads to formation of large blood-filled lesions localized mainly to the cervical region of skin. Whole-mount staining of the skin shown for the boxed region (below) demonstrating lymphatic identity (PROX1 expression) of the lesion forming cells and normal blood vasculature (EMCN staining) around the lesion. **c** Immunofluorescence staining of paraffin sections of embryonic back skin of E17 *PIK3CA*^{H1047R};*Vegfr3-CreER*^{T2} mice showing TER119^{+} red blood cells (RBC) inside (arrows) of the VEGFR3^{+} macrocystic lymphatic lesions. Dotted line indicates epidermis. **d-e** Characterization of *PIK3CA*^{H1047R}-driven microcystic LM. Whole mount immunofluorescence of back skin from an E17 *PIK3CA*^{H1047R};*Vegfr3-CreER*^{T2} embryo administered with 4-OHT (2 mg) at E14 (**d**). Antibodies and regions of skin are indicated. Lymphatic vessel branching is

quantified in (**e**). Data represent mean ± s.d. Two-tailed unpaired Student's *t*-test. Scale bars: 1 mm (**b**, **d**, whole skin), 200 µm (**b**, immunofluorescence), 50 µm (**c**), 250 µm (**d**). Source data are provided as a Source Data file.

a

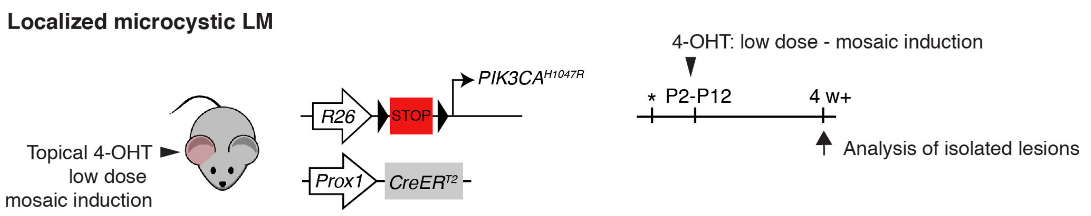

b

Dose	Stage	Analysis	Mice with LM	Untreated Ear	Treated Ear
1 μg	P2-P3	4 w	1/2 (50%)	0/1 (0%)	1/1 (100%)
2 μg	P2-P3	9 w[1]	1/4 (25%)	1/1 (100%)	1/1 (100%)
0.5 μg	P10-P12	8 w	3/3 (100%)	0/3 (0%)	3/3 (100%)
1 μg	P10-P12	8 w	1/2 (50%)	0/1 (0%)	1/1 (100%)
5 μg	P10-P12	8 w	8/8 (100%)	0/8 (0%)[2]	8/8 (100%)[2]

[1]Mouse with a lesion analyzed at 9 w (based on macroscopic observation of lesional bleeding), others at 27 w
[2]Based on macroscopic observation of lesional bleeding

c

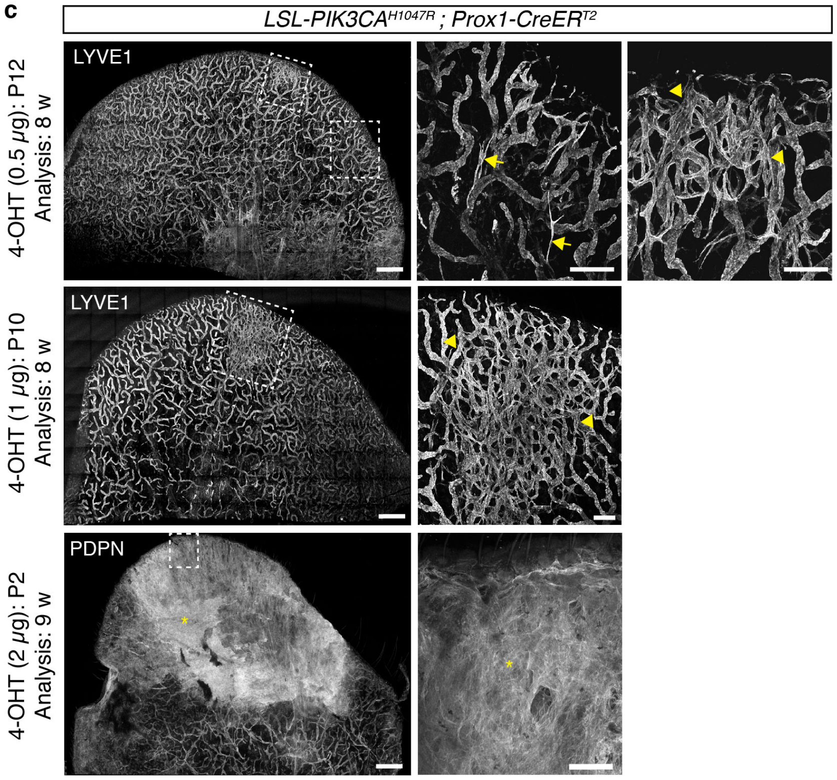

d

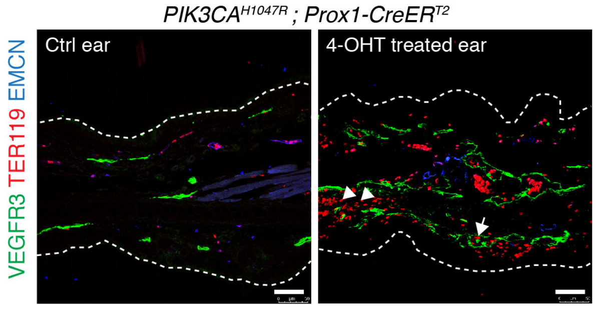

Supplementary Figure 3. Lymphatic overgrowth in *PIK3CA^{H1047R}*;*Prox1-CreER^{T2}* mice. a Genetic constructs and experimental plan for mosaic tamoxifen-inducible activation of *PIK3CA^{H1047R}* expression in lymphatic endothelia using the *ProxI-CreER^{T2}* mice. Asterisk indicates the date of birth. **b** Frequency of malformations observed after different 4-OHT

treatments in the *PIK3CA*^{H1047R};*Prox1-CreER*^{T2} mice. Data for both treated and untreated mice are shown. **c** Whole-mount immunofluorescence of ear skin of *PIK3CA*^{H1047R};*Prox1-CreER*^{T2} mice showing the progression of lymphatic overgrowth from initial sprouts (yellow arrows) to nodular lesions of hyperbranched vessels (yellow arrowheads) and to sheet-like lymphatic vascular structures (yellow asterisk). 4-OHT treatments and time points of analysis are indicated. Boxed areas are magnified on the left. **d** Immunofluorescence staining of paraffin sections of ear skin of 6-week old *PIK3CA*^{H1047R};*Prox1-CreER*^{T2} mouse topically administered with a low dose of 4-OHT (5 µg at P10). Note the presence of RBCs inside (arrows) and outside (arrowheads) of the VEGFR3^{+} lymphatic lesions. In control ear skin (*Ctrl;* untreated ear) RBCs are confined to the EMCN^{+} blood vessels. Dotted lines indicate epidermis. Scale bars: 1 mm (**c**, left panels), 200 µm (**c**, magnifications of boxed areas), 50 µm (**d**).

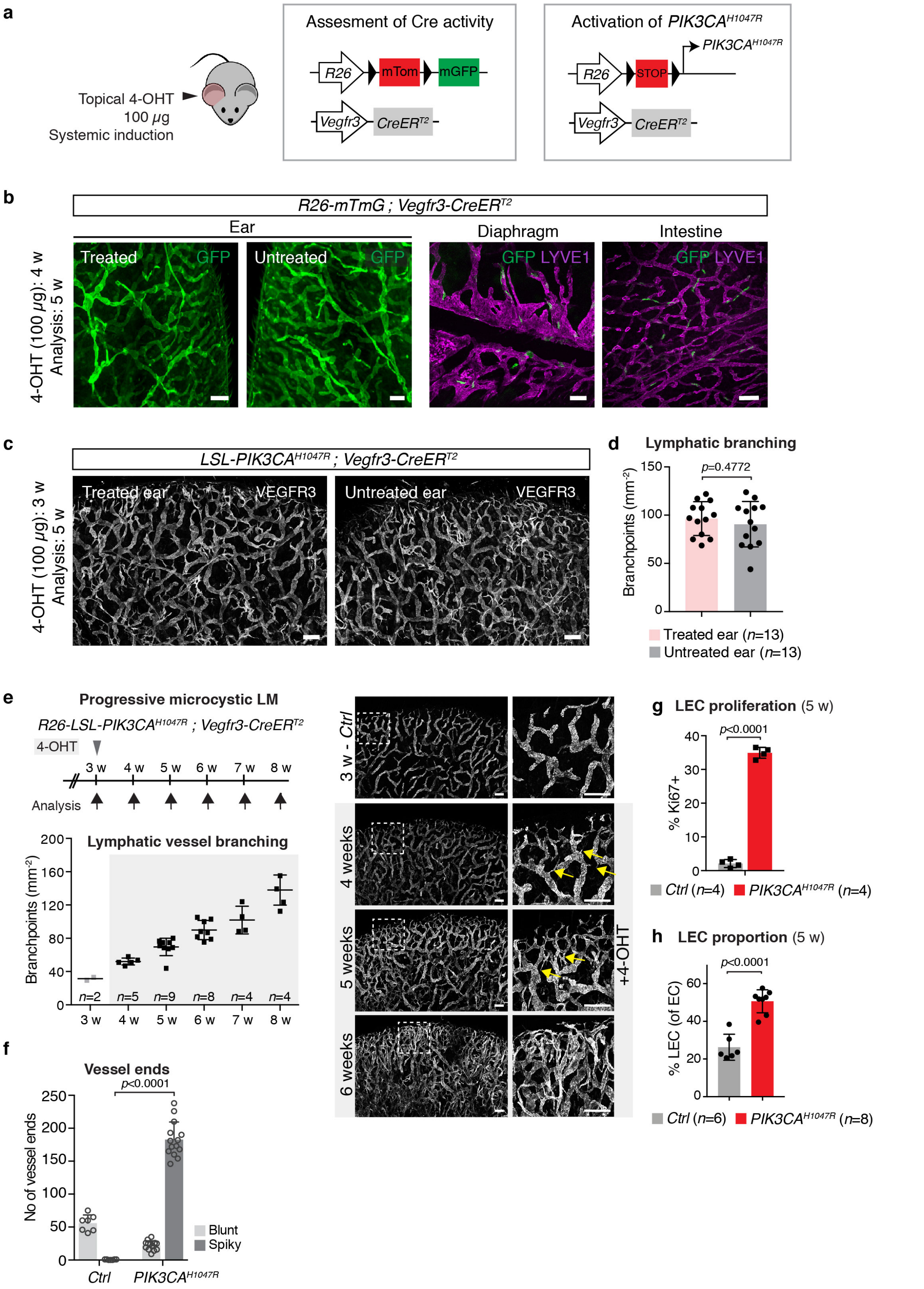

Supplementary Figure 4. Model of postnatal progressive microcystic LM. a Genetic constructs and experimental plan for systemic tamoxifen-inducible activation of Cre-reporter (left box) or *PIK3CA*^{H1047R} (right box) expression in lymphatic endothelia using the *Vegfr3-CreER*^{T2} mice. **b** GFP expression in a *R26-mTmG;Vegfr3-CreER*^{T2} mouse after topical application of 4-OHT to one ear. Note systemic recombination with high efficiency in both the

treated and untreated ears, likely reflecting strong VEGFR3 expression (translating into efficient Cre recombination) in dermal lymphatic vessels, and mosaic recombination in the intestine and diaphragm. **c, d** Analysis of lymphatic vessel hyperplasia in the *PIK3CA*^{H1047R}; *Vegfr3-CreER*^{T2} mice. Whole-mount immunofluorescence (**c**) and quantification of lymphatic vessel branching (**d**) showing no differences between 4-OHT-treated and untreated ears from the same mice. Data represent mean (*n*=13 mice) ± s.e.m. **e** Quantification of lymphatic vessel branching in *PIK3CA*^{H1047R};*Vegfr3-CreER*^{T2} mice after treatment with 4-OHT (100 µg) at P21 (*n*=2-10 ears as indicated) ± s.d. On the right: whole-mount immunofluorescence staining of ear skin showing induction of new sprouts (yellow arrows) in 4-OHT-treated mice. Boxed areas are magnified. **f** Quantification of the morphology of vessel ends in control (*n*=7) and *PIK3CA*^{H1047R} (*n*=14) mice. Data is included in Fig. 5a. **g**, **h,** Flow cytometry analysis of dermal LEC proliferation (**g**) and LEC numbers (proportion of PDPN^{+}PECAM1^{+} LECs of all PECAM1^{+} ECs) (**h**) in 5 weeks old 4-OHT-treated (*LSL-PIK3CA*^{H1047R}) and untreated (*Ctrl*) *PIK3CA*^{H1047R}; *Vegfr3-CreER*^{T2} mice (*n*=4). Data in (**d**, **f**, **g**, **h**) represent mean ± s.d. Two-tailed paired (**d**) or unpaired (**f**, **g**, **h**) Student's *t*-test. Scale bars: 200 µm. Source data are provided as a Source Data file.

a

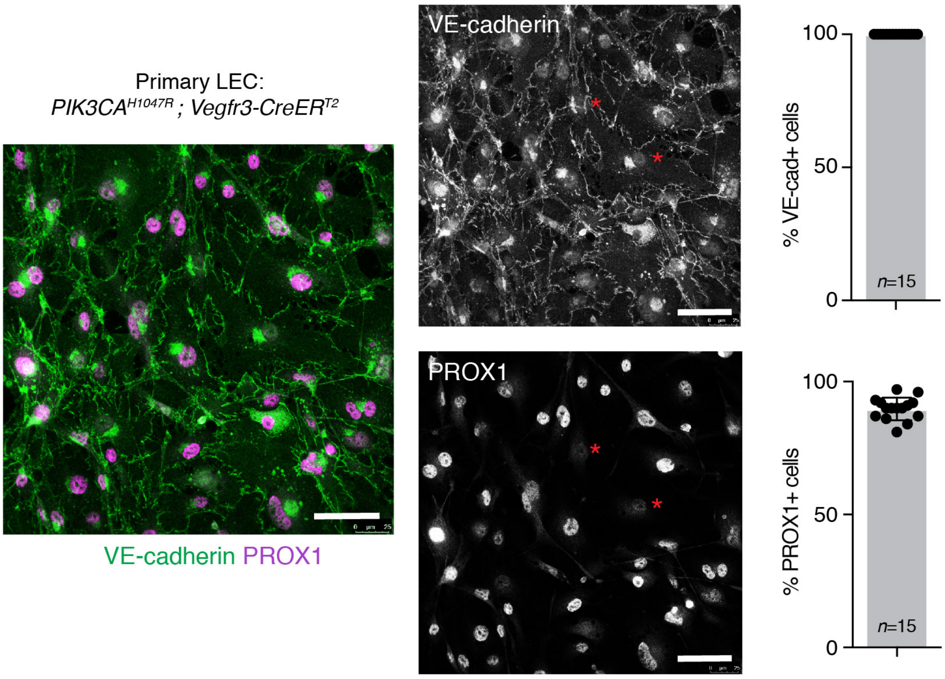

b

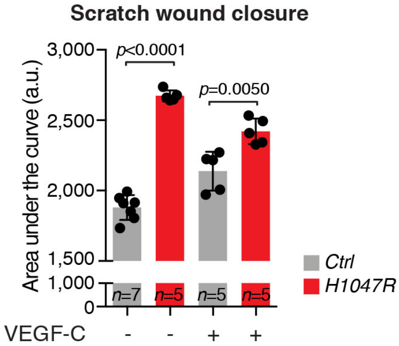

Supplementary Figure 5. Characterization of primary dermal LECs isolated from the *PIK3CA*^{H1047R}*; Vegfr3-CreER*^{T2} mice. a Quantification of VE-cadherin^{+} ECs and PROX1^{+} LECs in cultures of primary dermal ECs isolated by sequential selection with PECAM1 and LYVE1 antibody coated magnetic beads. Data is representative of 2 independent batches of cell isolation showing mean % of VE-cadherin^{+} cells (100 ± 0 %) and % of PROX1^{+} cells (89.7 ± 4.3 %) (*n*=15 biological replicates) ± s.d. Asterisks indicate a few VE-cadherin^{+}PROX1^{-} BECs (blood endothelial cells) present in the LEC population. **b** IncuCyte scratch assay showing faster wound closure in 4-OHT-treated (*PIK3CA*^{H1047R}-expressing) compared to vehicle-treated (*Ctrl*) primary LECs, but no further increase in the speed of wound closure in the *PIK3CA*^{H1047R} -expressing LECs in the presence of VEGF-C. Data is representative of 2 independent experiments showing mean wound closure rates, calculated as area under the curve (AUC) from the relative wound density (*n*=number of wells as indicated ± s.d.). Two-tailed unpaired Student's *t*-test. Scale bars: 25 µm. Source data are provided as a Source Data file.

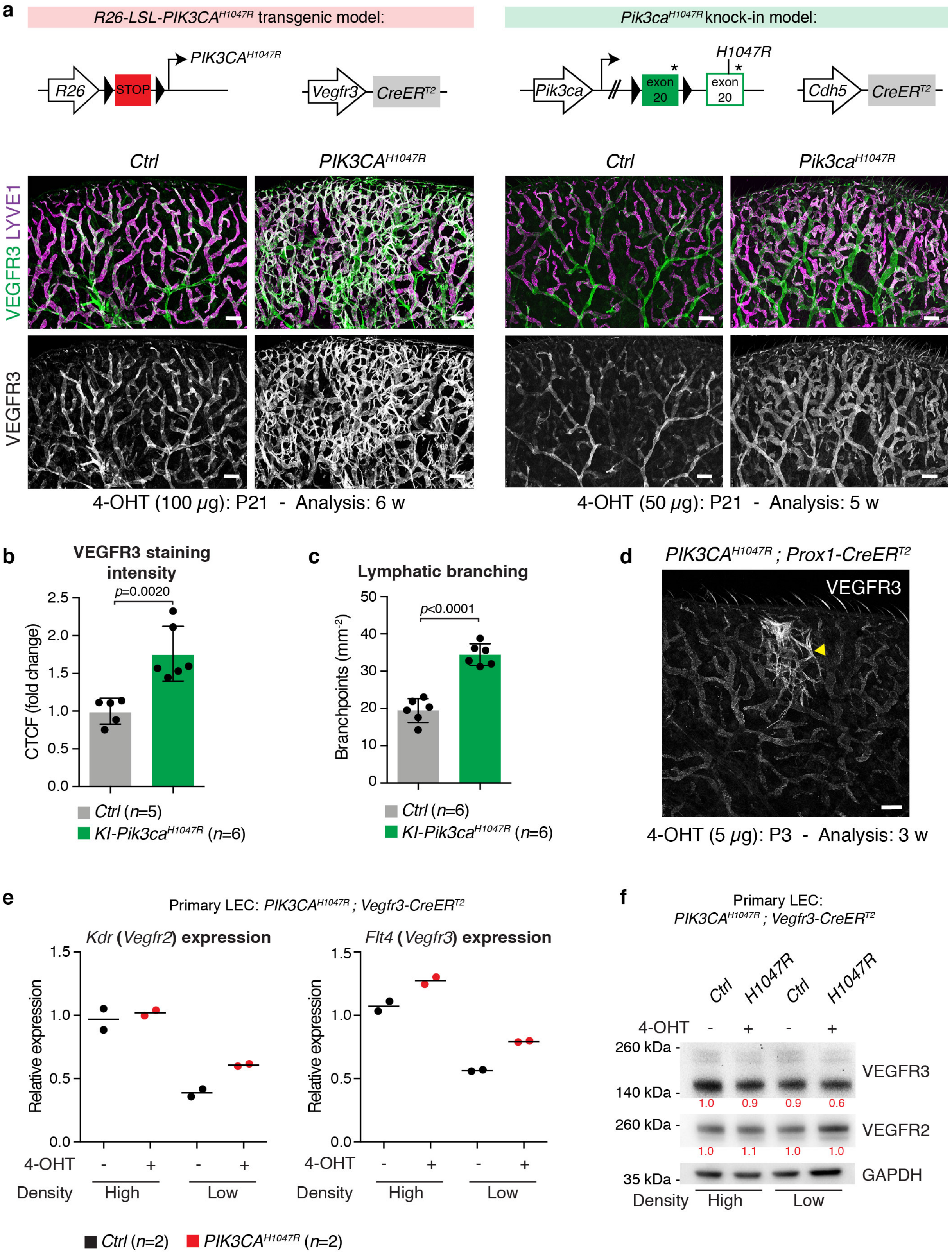

Supplementary Figure 6. VEGFR3 expression in *PIK3CA*^{H1047R} -driven microcystic LM. **a** Whole-mount immunofluorescence staining of ear skin of transgenic *LSL-PIK3CA*^{H1047R};*Vegfr3-CreER*^{T2} (left) and knock-in *KI-Pik3ca*^{H1047R};*Cdh5-CreER*^{T2} (right) mice, showing increase in VEGFR3 levels in the mutant ears. 4-OHT administration protocol and genetic constructs are indicated. Asterisk indicates stop codon. **b** Quantification of VEGFR3 staining intensity in the dermal lymphatic vasculature in 4-OHT-treated knock-in *KI-*

Pik3ca^{H1047R} ear skin, compared to littermate controls (*n*=5-6 mice as indicated). **c** Quantification of lymphatic vessel branching in *KI-Pik3ca*^{H1047R};*Cdh5-CreER*^{T2} mice treated as in (**a**) (*n*=6 mice). **d** Whole-mount immunofluorescence of ear skin of P21 *PIK3CA*^{H1047R};*Prox1-CreER*^{T2} mouse showing upregulation of VEGFR3 in a localized lesion (yellow arrowhead). Data in (**b, c**) represent mean ± s.d. **e, f** VEGFR2 and VEGFR3 expression analyzed by qRT-PCR (**e**) and western blot analysis (**f**) in primary LECs isolated from *PIK3CA*^{H1047R};*Vegfr3-CreER*^{T2} mice. No differences between 4-OHT-treated (*PIK3CA*^{H1047R}) compared to vehicle-treated (*Ctrl*) cells were detected either in high or low density cultures. The quantification of bands in the blots is shown in red. Data are from one experiment. Two-tailed unpaired Student's *t*-test. Scale bars: 200 µm. Source data are provided as a Source Data file.

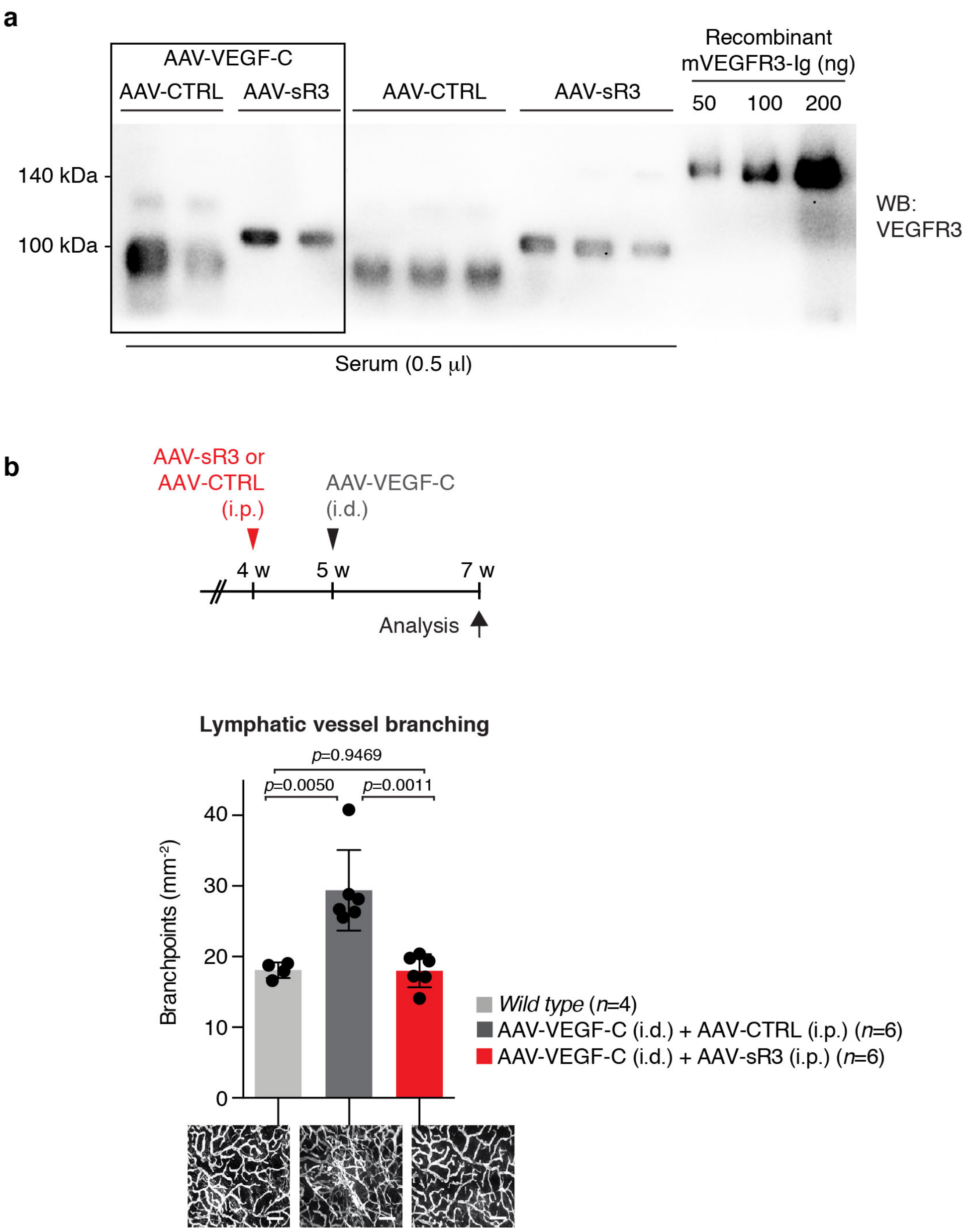

Supplementary Figure 7. Validation of VEGF-C neutralizing activity of systemically delivered AAV-VEGFR3$_{1\text{-}4}$-Ig in the ear skin. a Western blot analysis showing soluble VEGFR3$_{1\text{-}4}$-Ig or the control VEGFR3$_{4\text{-}7}$-Ig in serum 3 weeks after intraperitoneal injection of AAV. Data are representative of n=3 mice (AAV-CTRL and AAV-sR3 in the presence of AAV-VEGF-C) or n=9-11 mice (AAV-CTRL and AAV-sR3 in the absence of VEGF-C). **b** Experimental plan for validation of AAV-VEGFR3$_{1\text{-}4}$-Ig and quantification of lymphatic vessel branching in the ears of wild type mice and in mice receiving intradermal injection of AAV-VEGF-C with or without intraperitoneal AAV-sR3 injection 7 days earlier. Data represent mean (n=4-6 ears as indicated) ± s.d. Two-tailed unpaired Student's t-test. Scale bars: 250 µm. Source data are provided as a Source Data file.

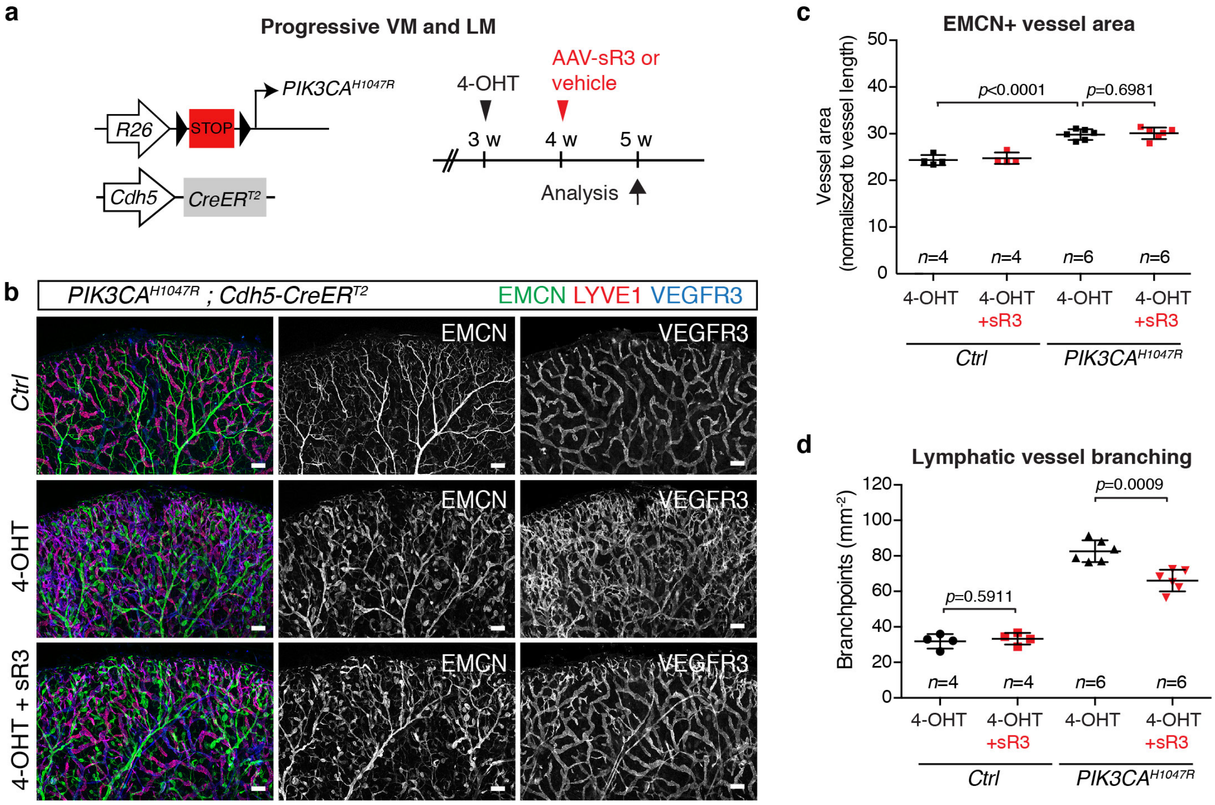

Supplementary Figure 8. Inhibition of VEGF-C signaling selectively inhibits lymphatic overgrowth in *PIK3CA*^{H1047R};*Cdh5-CreER*^{T2} mice. a Genetic constructs and experimental plan for the induction of both venous malformations (VM) and lymphatic malformations (LM) using the pan-endothelial specific *Cdh5-CreER*^{T2} mice, and treatment using the soluble VEGF-C trap (AAV-VEGFR3-Ig; AAV-sR3) or vehicle (PBS). **b** Whole-mount staining of ears from Cre^{-} littermate control mice (*Ctrl*) and *PIK3CA*^{H1047R};*Cdh5-CreER*^{T2} mice treated with 4-OHT (50 µg) and AAV-sR3 or vehicle. **c**,**d** Quantification of EMCN^{+} blood vessel area (**c**) and lymphatic vessel branching (**d**) in the control and AAV-sR3 treated (red symbols) *PIK3CA*^{H1047R};*Cdh5-CreER*^{T2} mice. Data represent mean ± s.d. Two-tailed unpaired Student's *t*-test. Scale bars: 200 µm (**b**). Source data are provided as a Source Data file.

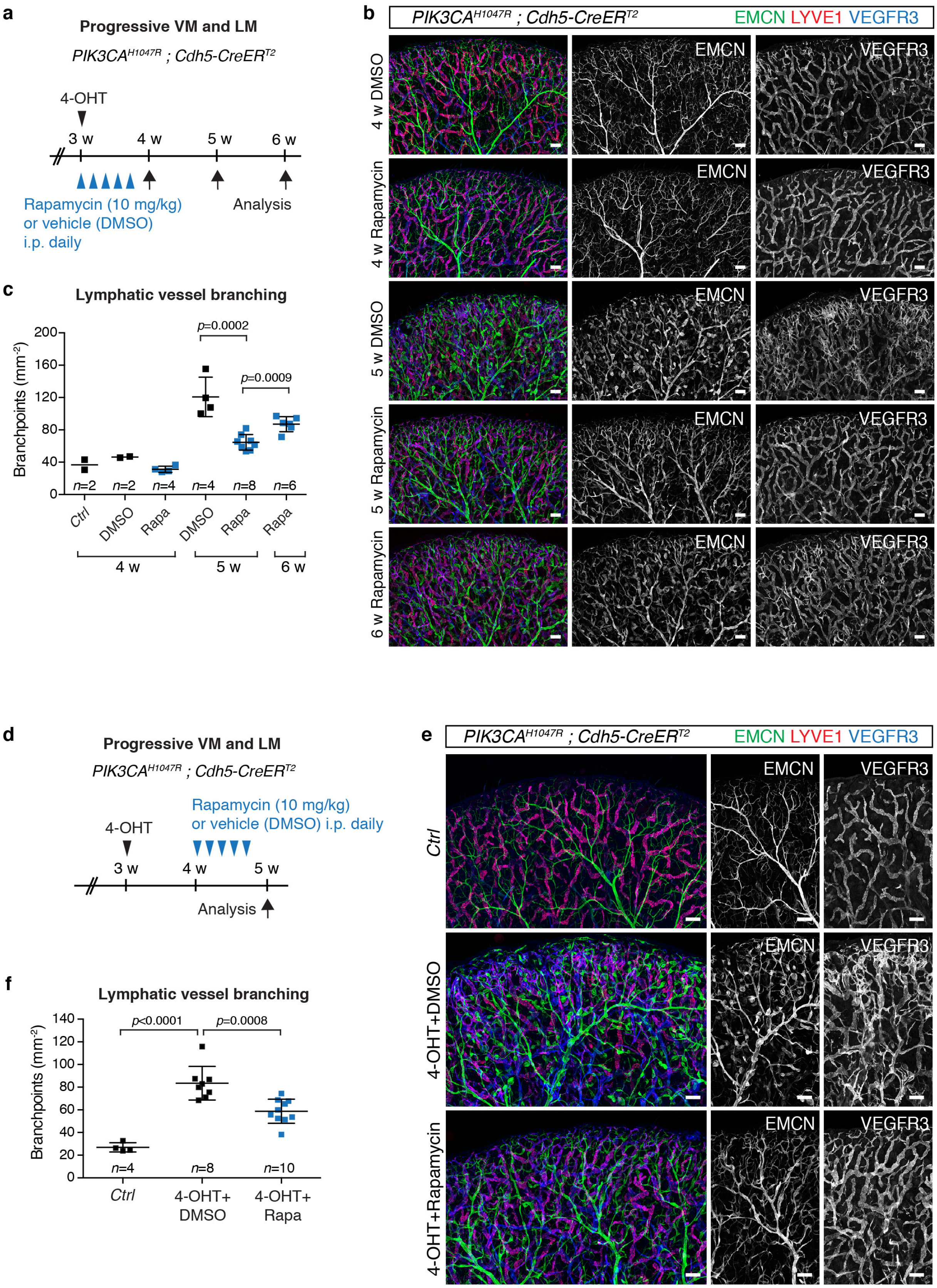

Supplementary Figure 9. Inhibition of the formation and growth of venous and lymphatic malformations by Rapamycin. a Experimental plan for the induction and Rapamycin prevention of VM and LM lesions in the *PIK3CA*^{H1047R};*Cdh5-CreER*^{T2} mice. **b** Whole mount immunofluorescence of ears from control mice (*Ctrl*) and 4-OHT (50 µg) treated

PIK3CA^{H1047R};*Cdh5-CreER*^{T2} mice treated with Rapamycin or the vehicle (DMSO). **c** Quantification of lymphatic vessel branching in the control and Rapamycin treated (blue squares) VM/LM mice. **d** Experimental plan for the induction and Rapamycin treatment of VM and LM lesions in the *PIK3CA*^{H1047R};*Cdh5-CreER*^{T2} mice. **e** Whole mount immunofluorescence of ears from Cre^{-} littermate mice (*Ctrl*) and 4-OHT treated *PIK3CA*^{H1047R};*Cdh5-CreER*^{T2} mice treated with Rapamycin or the vehicle (DMSO). **f** Quantification of lymphatic vessel branching in the control and Rapamycin treated (blue squares) VM/LM mice. Data in (**c**, **f**) represent mean (*n*=number of ears as indicated) ± s.d. Two-tailed unpaired Student's *t*-test. Scale bars: 200 µm (**b**, **e**). Source data are provided as a Source Data file.

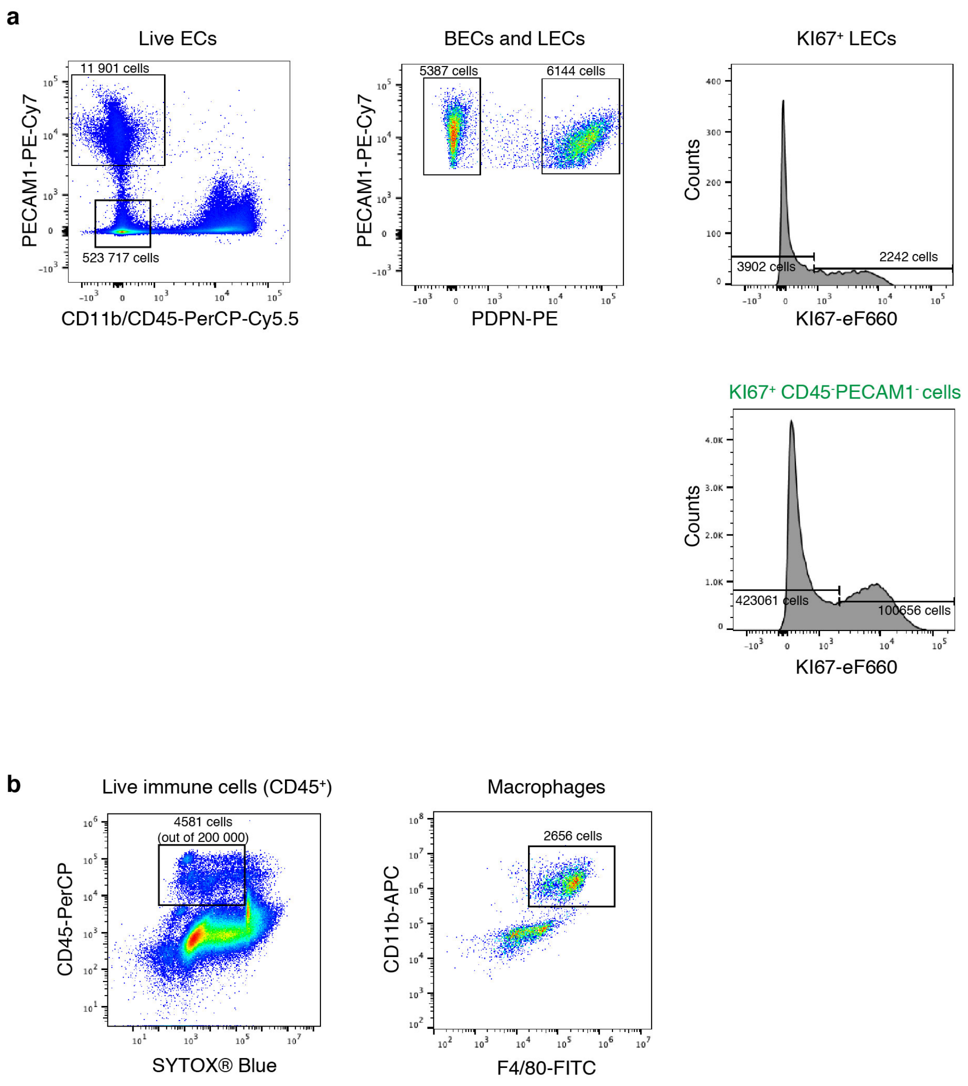

Supplementary Figure 10. Analysis of dermal LECs and macrophages by flow cytometry. Gating scheme for analysis of dermal ECs (**a**) and macrophages (**b**). Gating of KI67^{+} cells was based on analysis of non-immune / non-EC (CD45^{-}PECAM1^{-}) cells (below in **a**).
