# Supplementary material for: Blockade of VEGF-C signaling inhibits lymphatic malformations driven by oncogenic PIK3CA mutation
Source: Nat Commun. 2020 Jun 8;11:2869. doi: 10.1038/s41467-020-16496-y (PMC7280302; doi:10.1038/s41467-020-16496-y)
Supplement: Supplementary file 3 — Reporting Summary [file 41467_2020_16496_MOESM3_ESM.pdf]

nature research

Corresponding author(s): Taija Mäkinen

Last updated by author(s): Apr 29, 2020

Reporting Summary

Nature Research wishes to improve the reproducibility of the work that we publish. This form provides structure for consistency and transparency in reporting. For further information on Nature Research policies, see Authors & Referees and the Editorial Policy Checklist.

Statistics

For all statistical analyses, confirm that the following items are present in the figure legend, table legend, main text, or Methods section.

n/a	Confirmed	
☐	☒	The exact sample size (*n*) for each experimental group/condition, given as a discrete number and unit of measurement
☐	☒	A statement on whether measurements were taken from distinct samples or whether the same sample was measured repeatedly
☐	☒	The statistical test(s) used AND whether they are one- or two-sided<br>*Only common tests should be described solely by name; describe more complex techniques in the Methods section.*
☐	☒	A description of all covariates tested
☐	☒	A description of any assumptions or corrections, such as tests of normality and adjustment for multiple comparisons
☐	☒	A full description of the statistical parameters including central tendency (e.g. means) or other basic estimates (e.g. regression coefficient) AND variation (e.g. standard deviation) or associated estimates of uncertainty (e.g. confidence intervals)
☐	☒	For null hypothesis testing, the test statistic (e.g. F, t, r) with confidence intervals, effect sizes, degrees of freedom and P value noted<br>*Give P values as exact values whenever suitable.*
☒	☐	For Bayesian analysis, information on the choice of priors and Markov chain Monte Carlo settings
☒	☐	For hierarchical and complex designs, identification of the appropriate level for tests and full reporting of outcomes
☒	☐	Estimates of effect sizes (e.g. Cohen's *d*, Pearson's *r*), indicating how they were calculated

Our web collection on statistics for biologists contains articles on many of the points above.

Software and code

Policy information about availability of computer code

Data collection	Leica Application Suite X (Version 3.5.2.18963 and earlier) [image aquisition]<br>CytExpert (Version 1.0) (Beckman Coulter) [flox cytometry]<br>BD FACSDiva Software (Version 8.0) (BD Biosciences) [flow cytometry]<br>Image Lab Software (Version 4.1) [western blot]<br>IncuCyte ZOOMTM (Version 2016A) [scratch assay]<br>StepOneTM Software (version 2.3) [qRT-PCR]<br>Gen5 All-In-One Microplate Reader Software (Version 2.03.1) [protein concentration]<br>Highlander (Version 16.1) [variant filtering]
Data analysis	Image J (Version 2.0 or earlier)<br>Adobe Photoshop (Versions CS6, CC2018, CC2019)<br>Graphpad Prism 7.0<br>IncuCyteTM scratch wound cell migration software module (Version 2016A)<br>FlowJo 10.5.0-10.5.3 (TreeStar)

For manuscripts utilizing custom algorithms or software that are central to the research but not yet described in published literature, software must be made available to editors/reviewers. We strongly encourage code deposition in a community repository (e.g. GitHub). See the Nature Research guidelines for submitting code & software for further information.

Data

Policy information about availability of data

All manuscripts must include a data availability statement. This statement should provide the following information, where applicable:

- Accession codes, unique identifiers, or web links for publicly available datasets
- A list of figures that have associated raw data
- A description of any restrictions on data availability

The next-generation sequencing data that support the findings of this study are available on request from MV. The data are not publicly available due to them containing information that could compromise research participant consent. All other data supporting the findings of this study are available from the corresponding author (TM) upon request. The source data underlying Figs. 2c, 3b, f-i, 4b, d, g, 5c, d, f and Supplementary Figs. 2e, 4d-h, 5a, b, 6b, c, e-f, 7a, b, 8c, d, 9c, f are provided as a Source Data file. The PIK3CA c.3140A>G p.His1047Arg mutation is a well-known somatic mutation, already reported in various databases, including the large COSMIC database (the Catalogue Of Somatic Mutations In Cancers; ID:COSV55873195), in which it occurs 3360 times. Therefore, the mutation was not resubmitted.

Field-specific reporting

Please select the one below that is the best fit for your research. If you are not sure, read the appropriate sections before making your selection.

[x] Life sciences   [ ] Behavioural & social sciences   [ ] Ecological, evolutionary & environmental sciences

For a reference copy of the document with all sections, see nature.com/documents/nr-reporting-summary-flat.pdf

Life sciences study design

All studies must disclose on these points even when the disclosure is negative.

Sample size	Sample size was chosen in accordance with similar previously published experiments (e.g. Graupera et al, Nature 453, 662–666 (2008); Castillo et al. Sci. Transl. Med. 8, 332ra43 (2016); Baluk et al, JCI Insight 2, (2017)). Data shown in the main figures is based on a minimum of 3 mice per condition. In Supplementary Data, in a few instances the number of (wild type/untreated) controls, which are repeatedly included in other experiments, was <3 (Supp Fig 4e, 6e, 9c).
Data exclusions	The established vessel overgrowth phenotype was confirmed by immunofluorescence analysis in one ear of mutant mice when the other ear was used for FACS analysis (of EC proliferation, immune cell composition). In one experiment (Fig. 4d), one litter of Cre+ mice did not show the established Cre-mediated vessel overgrowth response (due to inefficient 4-OHT induction) and were excluded from the analysis.
Replication	Some data is from one experiment (with the indicated number of biological replicates included): Fig. 5d (n=4-5 mice), Supplementary Fig. 4g (n=4 mice), 6e (n=2 biological replicates), 7b (n=2-3 mice), 8c,d (n=2-3 mice). All other data has been successfully replicated in independent experiments and with mice from at least 2 separate litters. All the attempts at replication were successful except for one experiment (Fig. 4d), where one litter of Cre+ mice did not show the established Cre-mediated vascular overgrowth response (due to inefficient 4-OHT induction) and were excluded from the analysis (see previous point).
Randomization	Allocation of mice into experimental groups was based on genotype. Littermate controls were included. Both female and male mice were included in analyses, and no differences in the phenotype between the genders were observed.
Blinding	For most experiments, no blinding was done in the data collection, analysis and quantifications. However, quantification of vessel parameters (lymphatic vessel branching, immunofluorescence staining intensity) was done in an unbiased automated fashion using Angiotool plugin of ImageJ, or by measuring corrected total cell fluorescence in Image J. For quantification of vessel ends and the % of PROX1+/VE-cad+ cells in primary cultures was done manually using Photoshop software. Scratch assay was analyzed in an automated fashion using IncuCyteTM scratch wound cell migration software module.

Reporting for specific materials, systems and methods

We require information from authors about some types of materials, experimental systems and methods used in many studies. Here, indicate whether each material, system or method listed is relevant to your study. If you are not sure if a list item applies to your research, read the appropriate section before selecting a response.

Materials & experimental systems

n/a	Involved in the study	
[ ]	[x]	Antibodies
[x]	[ ]	Eukaryotic cell lines
[x]	[ ]	Palaeontology
[ ]	[x]	Animals and other organisms
[ ]	[x]	Human research participants
[x]	[ ]	Clinical data

Methods

n/a	Involved in the study	
[x]	[ ]	ChIP-seq
[ ]	[x]	Flow cytometry
[x]	[ ]	MRI-based neuroimaging

Antibodies

Antibodies used

The following antibodies were used for whole mount immunofluorescence (dilution 1:200-1:500): hamster anti-mouse PDPN (8.1.1-a, Developmental Studies Hybridoma Bank; 1:200), rat anti-mouse PECAM1 (553370, BectonDickinson; 1:200), rat anti-mouse LYVE1 (103-PA50AG, Reliatech; 1:500), rat anti-mouse EMCN (sc-65495, Santa Cruz; 1:200), goat anti-mouse NRP2 (AF567, R&D Systems; 1:200), goat anti-mouse VEGFR2 (AF644, R&D Systems;1:200), goat anti-mouse VEGFR3 (AF743, R&D Systems;1:200), rabbit anti-PROX1 (generated against human Prox1 C-terminus (567-737aa); Stanczuk et al, 2015; 1:200), rabbit anti-GFP (A11122, Thermo Fisher Scientific;1:200), chicken anti-GFP (ab13970, Abcam; 1:500), rabbit anti-pS6(Ser240/244) (cat2215, Cell Signaling Technology;1:200), goat anti-mouse VE-cadherin (cat AF1002, R&D Systems; 1:200). The following antibodies or reagents were used for immunostaining of cells: goat anti-VE-cadherin (sc-6458, Santa Cruz;1:100), Alexa FluorTM 568 Phallodin (A12380, Thermo Fisher;1:100), rabbit anti-PROX1 (generated against human Prox1 C-terminus (567-737aa), Stanczuk et al, 2015;1:200), goat anti-mouse VEGFR3 (cat AF743, R&D Systems;1:200), secondary antibodies conjugated to Cy3, Alexa Fluor 405, 488 or 647 (Jackson ImmunoResearch;1:300).

The following antibodies were used for immunostaining of mouse paraffin sections, together with TSA Amplification Kit (Perkin Elmer): goat anti-mouse VEGFR-3 (AF743, R&D Systems;1:300) and rat anti-mouse CD45 antibody [I3/2.3] (ab25386, Abcam;1:200), and after the TSA kit rat anti-mouse EMCN (sc-65495, Santa Cruz;1:200), rabbit anti-mouse LYVE1 (103-PA50AG, Reliatech;1:200) and Alexa Fluor® 647 anti-mouse TER-119/Erythroid Cells Antibody (116218, BioLegend;1:200). For human paraffin sections the following antibodies were used: rabbit anti-human LYVE1 (cat 102-PA50AG, Reliatech;1:100), rabbit anti-human PROX1 (cat 51043-1-AP, Proteintech Group;1:100), mouse anti-human PDPN (D2-40, cat M361901-2, Dako-Agilent;1:100), mouse anti-human VEGFR3 (9D9F9, cat MAB3757, Millipore;1:200), anti-Actin, alpha-Smooth muscle-Cy3 (A2547, Sigma;1:300) and rabbit anti-CD45 (HPA000440, Sigma;1:100).

The following antibodies were used for western blots: rabbit anti-mouse phospho-Akt (Ser473) (#4060), phospho-Akt (Thr308) (#2965), Akt (#9272), VEGFR2 (#2479), GAPDH (#2118, all from CST;1:2000), goat anti-mouse VEGFR3 (AF743, R&D Systems;1:1000), secondary antibodies conjugated to HRP (Jackson ImmunoResearch;1:5000).

For following antibodies were used for FACS: rat anti-mouse CD16/CD32 (eBioscience 14-0161-85;1:100), PDPN (8.1.1, PE, eBioscience 12-5381-81;1:300), CD31/PECAM1 (390, PerCP-eFluor 710, eBioscience 46-0311-80;1:300), CD45 (30-F11, PerCP-Cyanine5.5, eBioscience 45-0451-82;1:50), CD11b (M1/70, PerCP-Cyanine5.5, eBioscience 45-0112-82;1:50), PDPN (8.1.1, PE-Cyanine7, eBioscience 25-5381-82;1:300), CD31/PECAM1 (390, PE-Cyanine7, eBioscience 25-0311-82;1:100), CD45 (30-F11, eFluor 450, eBioscience 48-0451-82;1:50), CD11b (M1/70, eFluor 450, eBioscience 48-0112-82;1:50), Ki67 (SolA15, eFluor 660, eBioscience 50-5698-80;1:100), CD45 (30-F11, PerCP, BD Pharmingen- 557235;1:50), CD11b (M1/70, APC, eBioscience-17-0112-82;1:100), F4/80 (BM8, FITC, BioLegend-123108;1:50).

Antibody details and dilutions are provided in Supplementary Table 2.

Validation

The antibodies used in this study were validated for the species and applications by the indicated manufacturers. They have all been used in previous publications by us and others.

Antibodies used for immunostaining:
hamster anti-mouse PDPN: Developmental Studies Hybridoma Bank provides several references for validation. https://dshb.biology.uiowa.edu/8-1-1
rat anti-mouse PECAM1: BectonDickinson provides several references for validation. https://www.bdbiosciences.com/eu/applications/research/stem-cell-research/cancer-research/mouse/purified-rat-anti-mouse-cd31-mec-133/p/553370
rabbit anti-mouse LYVE1: Reliatech provides several references for validation. https://www.reliatech.de/products/antibodies/polyclonal-antibodies/product/103-pa50ag/
rat anti-mouse EMCN: Santa Cruz Biotechnology provides several references for validation. https://www.scbt.com/p/endomucin-antibody-v-7c7
goat anti-mouse NRP2: R&D Systems provides several references for validation. https://www.rndsystems.com/products/mouse-rat-neuropilin-2-antibody_af567
goat anti-mouse VEGFR2: R&D Systems provides several references for validation. https://www.rndsystems.com/products/mouse-vegfr2-kdr-flk-1-antibody_af644
goat anti-mouse VEGFR3: R&D Systems provides several references for validation. https://www.rndsystems.com/products/mouse-vegfr3-flt-4-antibody_af743
rabbit anti-GFP: Thermo Fisher Scientific provides several references for validation. https://www.thermofisher.com/antibody/product/GFP-Antibody-Polyclonal/A-11122
chicken anti-GFP: Abcam provides several references for validation. https://www.abcam.com/gfp-antibody-ab13970.html
rabbit anti-pS6 (Ser240/244): Cell Signaling Technology provides several references for validation. https://www.cellsignal.com/products/primary-antibodies/phospho-s6-ribosomal-protein-ser240-244-antibody/2215
goat anti-mouse VE-cadherin: R&D Systems provides several references for validation. https://www.rndsystems.com/products/mouse-ve-cadherin-antibody_af1002
goat anti-VE-cadherin: Santa Cruz Biotechnology provides several references for validation. https://www.scbt.com/p/ve-cadherin-antibody-c-19
AF568 Phalloidin: Thermo Fisher Scientific provides several references for validation. http://www.thermofisher.com/order/catalog/product/A12380
rat anti-mouse CD45 [I3/2.3] : Abcam provides several references for validation. https://www.abcam.com/cd45-antibody-i323-ab25386.html
anti-mouse TER-119 AF647: BioLegend provides several references for validation. https://www.biolegend.com/en-us/products/alexa-fluor-647-anti-mouse-ter-119-erythroid-cells-antibody-3277
mouse anti-human VEGFR3 (9D9F9): Millipore provides several references for validation. https://www.antibodypedia.com/gene/3956/VEGFA/antibody/551444/MAB3757
anti-Actin, alpha-Smooth muscle Cy3: Sigma provides several references for validation. https://www.sigmaaldrich.com/catalog/

product/sigma/a2547?lang=en&region=SE
rabbit anti-human CD45: Sigma provides several references for validation. https://www.sigmaaldrich.com/catalog/product/sigma/hpa000440?lang=en&region=US
rabbit anti-human LYVE1: Reliatech provides several references for validation. https://www.reliatech.de/products/antibodies/polyclonal-antibodies/product/102-pa50ag/
rabbit anti-human PROX1: Proteintech Group provides several references for validation. https://www.ptglab.com/products/PROX1-Antibody-51043-1-AP.htm
mouse anti-human PDPN (D2-40): Dako-Agilent provides several references for validation. https://www.agilent.com/store/en_US/Prod-M361901-2/M361901-2
rabbit anti-PROX1: generated against human Prox1 C-terminus (567-737aa) and validated in the laboratory based on nuclear immunofluorescence staining pattern in LECs. It has been used in several publications, e.g. Stanczuk et al. Cell Rep. (2015).

Antibodies used for flow cytometry:
rat anti-mouse CD16/CD32: eBioscience provides several references for validation. https://www.thermofisher.com/antibody/product/CD16-CD32-Antibody-clone-93-Monoclonal/14-0161-82
PDPN (8.1.1, PE): eBioscience provides several references for validation. https://www.thermofisher.com/antibody/product/Podoplanin-Antibody-clone-eBio8-1-1-8-1-1-Monoclonal/12-5381-82
CD31/PECAM1 (390, PE-Cyanine7): eBioscience provides several references for validation. https://www.thermofisher.com/antibody/product/CD31-PECAM-1-Antibody-clone-390-Monoclonal/25-0311-82
CD45 (30-F11, PerCP-Cyanine5.5): eBioscience provides several references for validation. https://www.thermofisher.com/antibody/product/CD45-Antibody-clone-30-F11-Monoclonal/45-0451-82
CD11b (M1/70, PerCP-Cyanine5.5): eBioscience provides several references for validation. https://www.thermofisher.com/antibody/product/CD11b-Antibody-clone-M1-70-Monoclonal/45-0112-82
PDPN (8.1.1, PE-Cyanine7): eBioscience provides several references for validation. https://www.thermofisher.com/antibody/product/Podoplanin-Antibody-clone-eBio8-1-1-8-1-1-Monoclonal/25-5381-82
CD31/PECAM1 (390, PerCP-eFluor 710): eBioscience provides several references for validation. https://www.thermofisher.com/antibody/product/CD31-PECAM-1-Antibody-clone-390-Monoclonal/46-0311-82
CD45 (30-F11, eFluor 450): eBioscience provides several references for validation. https://www.thermofisher.com/antibody/product/CD45-Antibody-clone-30-F11-Monoclonal/48-0451-82
CD11b (M1/70, eFluor 450): eBioscience provides several references for validation. https://www.thermofisher.com/antibody/product/CD11b-Antibody-clone-M1-70-Monoclonal/48-0112-82
Ki67 (SolA15, eFluor 660): eBioscience provides several references for validation. https://www.thermofisher.com/antibody/product/Ki-67-Antibody-clone-SolA15-Monoclonal/50-5698-82
CD45 (30-F11, PerCP): eBioscience provides several references for validation. https://www.bdbiosciences.com/us/applications/research/stem-cell-research/cancer-research/mouse/percp-rat-anti-mouse-cd45-30-f11/p/557235
CD11b (M1/70, APC): eBioscience provides several references for validation. https://www.thermofisher.com/antibody/product/CD11b-Antibody-clone-M1-70-Monoclonal/17-0112-82
F4/80 (BM8, FITC): BioLegend provides several references for validation. https://www.biolegend.com/en-us/products/fitc-anti-mouse-f4-80-antibody-4067

Antibodies used for western blot:
rabbit anti-mouse phospho-Akt (Ser473): Cell Signaling Technology provides several references for validation. https://www.cellsignal.com/products/primary-antibodies/phospho-akt-ser473-d9e-xp-rabbit-mab/4060
phospho-Akt (Thr308): Cell Signaling Technology provides several references for validation. https://www.cellsignal.com/products/primary-antibodies/phospho-akt-thr308-c31e5e-rabbit-mab/2965
Akt: Cell Signaling Technology provides several references for validation. https://www.cellsignal.com/products/primary-antibodies/akt-antibody/9272
VEGFR2: Cell Signaling Technology provides several references for validation. https://www.cellsignal.com/products/primary-antibodies/vegf-receptor-2-55b11-rabbit-mab/2479
GAPDH: Cell Signaling Technology provides several references for validation. https://www.cellsignal.com/products/primary-antibodies/gapdh-14c10-rabbit-mab/2118

Animals and other organisms

Policy information about studies involving animals; ARRIVE guidelines recommended for reporting animal research

Laboratory animals	R26-LSL-Pik3caH1047R (Eser et al, 2013), knock-in Pik3caH1047 (Kinross et al, 2012), Cdh5-CreERT2 (Wang et al, 2010), Vegfr3-CreERT2 (Martinez-Corral et al, 2016), Prox1-CreERT2 (Bazigou et al, 2011) and R26-mTmG (Muzumdar et al, 2007) mice were analyzed on a C57BL/6J background. Both female and male mice were used for analysis and no differences in the phenotype between the genders were observed. Both embryonic (E11-E17) and postnatal (P0-8 weeks) were used for experiments. The stage/age is stated in the figures and/or legends. Mice were housed in individually ventilated cages under a 12:12-h dark–light cycle (light from 07:00 to 19:00) at 22 ± 1°C with ad libitum access to food and water.
Wild animals	The study did not involve wild animals.
Field-collected samples	The study did not involve samples collected from the field.
Ethics oversight	All experimental procedures were approved by the Uppsala Animal Experiment Ethics Board and performed in compliance with all relevant Swedish regulations, or the Catalan Departament d' Agricultura, Ramaderia i Pesca, following protocols approved by the local Ethics Committees of IDIBELL-CEEA.

Note that full information on the approval of the study protocol must also be provided in the manuscript.

Human research participants

Policy information about studies involving human research participants

Population characteristics	Clinical features of patients with microcystic (3 males; sample collected for analysis during surgical resection of the lesion at 2, 6, 13 years of age) and macrocystic (1 male; sample collected for analysis during surgical resection at 27 months of age, 1 female; surgical resection at 7 years of age) lymphatic malformation included in the study are summarized in Table 1. All patients had a somatic p.H1047R mutation.
Recruitment	Residual tissue of macrocytic and microcystic LMs were collected from patients undergoing therapeutical surgery. As part of a clinical routine, the physician in charge decides on clinical basis whether surgery is used as therapy or not. When surgery is performed, the resected tissues are systematically screened for PIK3CA mutations. Within those, we looked for lesions with the H1047R mutation and either microcystic or macrocystic phenotype, of which five representative lesions (3 microcystic and 2 macrocystic) were selected for the study. There is no identifiable bias with the approach.
Ethics oversight	Studies were approved by the ethical committee of the Medical Faculty of the University of Louvain, Brussels, Belgium. All protocols were compliant with the Belgian laws governing research on human subjects.

Note that full information on the approval of the study protocol must also be provided in the manuscript.

Flow Cytometry

Plots

Confirm that:

☒ The axis labels state the marker and fluorochrome used (e.g. CD4-FITC).

☒ The axis scales are clearly visible. Include numbers along axes only for bottom left plot of group (a 'group' is an analysis of identical markers).

☒ All plots are contour plots with outliers or pseudocolor plots.

☒ A numerical value for number of cells or percentage (with statistics) is provided.

Methodology

Sample preparation	For FACS analysis of proliferating cells ear skin of adult mice were dissected, cut into small pieces and digested in Collagenase IV (Life Technologies) 10 mg/ml, DNase1 (Roche) 0.1 mg/ml and FBS 0.5 % (Life Technologies) in PBS at 37 °C for 30 min. Collagenase activity was quenched by dilution with FACS buffer (PBS, 0.5 % FBS, 2 mM EDTA) and digestion products were filtered twice through 70 µm nylon filters (BD Biosciences). Cells were washed with FACS buffer and immediately processed for immunostaining first by blocking Fc receptor binding with rat anti-mouse CD16/CD32 followed by incubation with antibodies targeting PDPN, CD31/PECAM1, CD45 and CD11b. After staining, cells were washed with PBS and then stained for dead cells using the blue LIVE/DEAD® fixable dead cell stain kit (Life Technologies), followed by fixation and permeabilization using the Foxp3/Transcription factor staining kit according to the manufacturer's instructions. Finally cells were incubated with rat serum and KI67 antibody. For FACS analysis of immune cells the ear skin was dissected into dorsal and ventral parts, cut into pieces and digested in Liberase TL (Roche) 100 µg/ml, Dnase1 (Roche) 0.5 mg/ml (Life Technologies) in PBS with 0.2% FBS at 37 °C for 1.5 h at 500 rpm. Liberase TL activity was quenched by adding 2 mM EDTA and the product was filtered through 50 µm filters (CellTricks). The cells were washed with FACS buffer (PBS, 0.5% FBS, 2 mM EDTA) and incubated first with rat anti-mouse CD16/32 antibody for blocking of the Fc receptor binding and then with CD45, CD11b and F4/80 targeting antibodies. Cell death was analyzed by adding SYTOX Blue dead stain (Life Technologies). The cell suspension was filtered again immediately before analysis.
Instrument	Cells were analyzed on a BD LSR Fortessa cell analyzer equipped with 5 lasers (355, 405, 488, 561 and 643 nm), or CytoFLEX Flow Cytometer (Beckman Coulter) with 4 lasers (405, 488, 561 and 633 nm).
Software	FlowJo software version 10.5.0-10.5.3 (TreeStar)
Cell population abundance	Analysis of proliferating LECs: ECs (PECAM1+) of total cells 1.5-2%; LECs (PDPN+) of ECs 20-30% (Ctrl) or 40-50% (Mutant), KI67+ LECs of all LECs 1-3% (Ctrl) or 15-40% (Mutant).<br>Analysis of immune cells: 200 000 cells were sorted, CD45+ viable cells about 5% of total cells, CD11b+F4/80+ cells of CD45+ cells 30-60% depending on the genotype and treatment.
Gating strategy	Single viable cells were gated from FSC-A/SSC-A, FSC-H/FSC-W and SSC-H/SSC-W plots followed by exclusion of dead cells in the UV dump channel. FMO controls were used to set up the subsequent gating scheme to obtain cell populations and quantification of proliferating cells. For analysis of immune cells, single cells were gated from SSC-H/FSC-H and FSC-Width/FSC-H plots followed by gating for CD45+ viable cells and CD11b+F4/80+ cells.

☒ Tick this box to confirm that a figure exemplifying the gating strategy is provided in the Supplementary Information.
